# Psychotic Experiences and Risk of Violence Perpetration and Arrest in the General Population: A Prospective Study

**DOI:** 10.1371/journal.pone.0159023

**Published:** 2016-07-22

**Authors:** Steven Honings, Marjan Drukker, Margreet ten Have, Ron de Graaf, Saskia van Dorsselaer, Jim van Os

**Affiliations:** 1 Department of Psychiatry and Psychology, South Limburg Mental Health Research and Teaching Network, Maastricht University Medical Centre, Maastricht, the Netherlands; 2 Netherlands Institute of Mental Health and Addiction, Utrecht, the Netherlands; 3 King's College London, King's Health Partners, Department of Psychosis Studies, Institute of Psychiatry, London, United Kingdom; King's College London, UNITED KINGDOM

## Abstract

**Background:**

In cross-sectional, general population studies, psychotic experiences have been associated with an increased risk of physical violence perpetration and arrest. However, longitudinal research on this topic is lacking. Moreover, it remains unclear whether subjects with psychotic experiences are also at risk of displaying psychological violence. The present study aims to investigate these associations.

**Method:**

The longitudinal association between baseline psychotic experiences and six-year incidence of violence perpetration and three-year incidence of arrest was studied in a prospective cohort of 6646 general population adults. Logistic regression analyses with varying levels of adjustment were performed in the complete sample and in subsamples stratified by presence or absence of baseline mental disorders.

**Results:**

The presence of psychotic experiences at baseline increased the risk of physical violence, psychological violence and arrest at follow-up. However, adjustment for dimensional measures of psychopathology and contextual confounders reduced all associations considerably. After adjustment, both clinically validated (OR = 3.59, 95% CI 1.09–11.81) and self-reported hallucinations (OR = 2.83, 95% CI 1.05 7.65) remained significantly associated with physical violence perpetration. Self-reported (OR = 3.06, 95% CI 1.55–6.03) and clinically validated delusions (OR = 3.24, 95% CI 1.47–7.13) were associated with an increased risk of arrest. There was no significant association between psychotic experiences and incident psychological violence in the fully adjusted model.

**Conclusion:**

Specific psychotic experiences may differentially predict physical violence perpetration and arrest, even after adjustment for demographics, dimensional measures of psychopathology and contextual confounders. However, more longitudinal research with larger sample sizes is required to confirm these findings.

## Introduction

Psychotic experiences (PE) are hallucinatory or delusional experiences falling below the threshold of a diagnosable psychotic disorder [1, 2]. In the general population, PE have been associated with an increased risk of violence perpetration [3–7] and arrest [4]. However, the temporal sequence remains unclear since longitudinal studies on this topic are lacking. Moreover, it remains unclear whether individuals with PE also have an increased risk of displaying psychological violence.

For many years, researchers have studied the association between mental disorders and the risk of violent behaviour [8–10]. Studies show that individuals with various major mental disorders including psychotic disorders are at increased risk of violence perpetration [11–17]. This link has been shown in both offender populations [18] and general population samples [19, 20]. However, the association between mental disorders and violence is likely to be confounded by several factors, including comorbid substance use [21] and contextual confounders like negative life events and low social support [20]. Moreover, the proportion of community violence related to mental disorders varies between 3% and 20%, indicating that the vast majority of community violence cannot be attributed to mental disorders [19, 22, 23].

In contrast to the literature on violence and full-blown psychosis [11–13], studies on the association between low grade PE and violence are scarce. Cross-sectional studies have shown that PE in the general population are associated with an increased risk of interpersonal violence [3–7], violence towards objects [5] and arrest [4]. Moreover, studies in detained youth showed that PE are more prevalent among incarcerated adolescents than among adolescents in the general population [24]. Similarly, violent incarcerated boys showed significantly more psychotic symptomatology than less violent incarcerated boys [25].

Previous studies on the association between PE and violent behaviour, while instructive, had some methodological limitations. First, all studies used cross-sectional study designs to investigate the association [3–7], thus making it impossible to infer the temporal sequence, which is required to verify the hypothesis that PE induce violence perpetration, rather than vice versa. Second, no studies adjusted the analyses for dimensional measures of psychopathology. Instead, adjustments were made for various dichotomously defined risk factors associated with violent behaviour, including substance use and other mental disorders, thus leaving room for residual confounding [3–7]. Third, studies used self-reported PE as their independent variable rather than clinically validated PE [3–7]. Although studies have shown that self-report questionnaires can detect PE with high accuracy [26], clinical interviews with sufficient PE items provide the most reliable estimate [1]. Finally, no previous study investigated the associations between PE and forms of violent behaviour other than physical violence, for example psychological violence, which has been similarly linked to various mental disorders [20].

The present study aimed to examine the longitudinal association between both self-reported and clinically validated PE on the one hand, and incident physical violence perpetration, psychological violence perpetration and arrest on the other, using data from a prospective, general population cohort. Moreover, it assessed to what extent the association may be confounded by non-psychotic psychopathology both at the level of dimensional symptom scores and the level of mental disorders as defined in the fourth edition of the Diagnostic and Statistical Manual of Mental Disorders (DSM-IV). Confounding by categorical mental disorder was examined using the method of stratification by presence or absence of baseline mental disorders. The influence of other demographic and contextual confounders identified in the literature was also examined. Finally, the present study analysed to what degree self-reported PE were similarly predictive of violent behaviour as clinically validated PE.

It was hypothesized that (i) PE would be longitudinally associated with an increased risk of incident physical violence, psychological violence and arrest; (ii) this association would be confounded by co-occurring non-psychotic psychopathology that is highly prevalent among subjects with PE [27–29]; (iii) a degree of association between PE and violence and arrest would remain even after taking into account confounding [20]; (iv) self-reported PE would be equally predictive of violence and arrest as clinically validated PE.

## Methods

### Sample

This study uses data of the second Netherlands Mental Health Survey and Incidence Study (NEMESIS-2), a longitudinal study of the prevalence, incidence, course and consequences of mental disorders in the Dutch general population [30]. NEMESIS-2 was approved by the Medical Ethics Review Committee for Institutions on Mental Health Care (METIGG). Participants provided written informed consent to participate in the interview, after full written and verbal information about the study was given before and at the start of the baseline assessment. Participants were selected based on a multistage random sampling procedure. In the first wave (T0), a total of 6646 persons aged 18–64 years were included. Participants were approached for two follow-up surveys, respectively three years (T1) and six years (T2) after baseline. At T1, 5503 persons were interviewed again (response rate 83%). At T2, 4618 persons were interviewed (response rate 69%). The mean period between the baseline interview and second follow-up interview was 6 years and 6 days. A more comprehensive description of the design can be found elsewhere [30].

### Diagnostic instrument

Participants were interviewed using the Composite International Diagnostic Interview Version 3.0 (CIDI 3.0), a fully structured lay-administered diagnostic interview generating DSM-IV diagnoses [31]. An important difference between the CIDI 3.0 and earlier versions is that the CIDI 3.0 contains a screening section consisting of key questions for most mental disorders. Only when a participant answers positively to a key question of the screening section, the complete CIDI section for that mental disorder is administered. The disorders considered in this study included mood disorders (major depression, dysthymia, bipolar disorder), anxiety disorders (panic disorder, agora-phobia without panic disorder, social phobia, specific phobia, generalized anxiety disorder), substance use disorders (alcohol/drug abuse and dependence) and impulse-control disorders (attention deficit hyperactivity disorder, conduct disorder, oppositional defiant disorder). Furthermore, antisocial personality disorder was measured using questions from the International Personality Disorder Examination [32], which are part of CIDI 3.0 [33, 34]. Impulse-control disorders were only assessed in subjects aged 18–44 years because of the possibility of recall bias in older participants [35]. All other mental disorders were assessed in all participants. Clinical calibration studies in various countries reported that the CIDI 3.0 assesses mood disorders (area under ROC curve (AUC) ranging between 0.75–0.93), anxiety disorders (AUC = 0.73) and substance use disorders (AUC 0.62–0.88) with good validity when clinical reappraisal interviews are used as the gold standard [36]. In addition, a clinical reappraisal interview carried out in a subsample of the National Comorbidity Survey Replication performed in the USA also found a valid assessment of ADHD in the CIDI 3.0 [37]. For the present analyses, lifetime diagnoses at baseline were used.

### Psychotic experiences at baseline

Baseline psychotic experiences were assessed using a psychosis add-on instrument based on the section of psychotic symptoms in CIDI 1.1. The instrument consisted of 20 questions regarding lifetime psychotic experiences. The 20 items consisted of 15 delusional experiences and 5 hallucinatory experiences, described in detail elsewhere [38]. Individuals who reported at least one lifetime psychotic experience were contacted for clinical re-interview over the telephone by an experienced clinician at the level of psychologist or psychiatrist within 8 weeks after the initial interview. Re-interview took place with questions from the Structured Clinical Interview for DSM-IV, which assesses psychotic disorders with good reliability [39, 40]. Findings from all re-interviews were discussed with a second clinician. Diagnoses of psychotic disorder were based on the data from the clinical re-interviews. Clinically validated PE were considered present when the psychotic nature of the self-reported psychotic experiences was confirmed at the clinical re-interview. Of all participants eligible for re-interview, 74% was actually re-interviewed. There were no large or significant differences between individuals who participated in the clinical re-interview and those who did not with regard to demographics, baseline mental disorders [41], violence perpetration or arrest. For the present study, both self-reported PE and clinically validated PE were used. Self-reported or clinically validated PE were considered present if individuals reported any self-reported or clinically validated CIDI psychotic symptom, respectively. In addition, continuous scales of both self-reported and clinically validated PE were computed. Individuals with psychotic disorder at baseline were excluded in order to ensure analyses reflected effects of subclinical PE.

### Violence perpetration and arrest at baseline and follow-up

Perpetration of both physical violence and psychological violence was assessed at baseline by asking participants about lifetime physical or psychological violence towards one of their current or former intimate partners or towards any of their children during upbringing. To increase the likelihood of these acts being reported, they were not mentioned as such but were listed in a booklet (lists A and B) and referred to by number. The items on list A referred to psychological violence and included name-calling, offending, belittling, punishing unjustly, blackmailing and threatening. The items on list B concerned physical violence and included kicking, biting, hitting, trying to wound with an object (gun, knife, piece of wood, pair of scissors, other object) or hot water. An additional question from the CIDI screener section was used to assess baseline physical violence by asking: “Have you ever had attacks of anger when all of a sudden you lost control and hit or tried to hurt someone?” At both follow-up interviews (T1, T2), participants were asked about physical and psychological violence between waves by using the same lists as at baseline. In contrast to baseline, violence at follow-up included acts against any person in general. To reduce the effects of this discrepancy, participants without a partner or children at baseline were excluded when analysing physical or psychological violence. Consistent with previous studies, psychological violence was defined as present if it occurred on two or more occasions and physical violence on one or more occasions [20, 42, 43]. Incident psychological violence perpetration (hereafter: psychological violence) and incident physical violence perpetration (hereafter: physical violence) were defined as present if the participant displayed the respective violent behaviour at any of the follow-up surveys, while subjects with the respective violent behaviour at baseline were excluded.

Self-reported arrest was obtained at baseline (T0) and at the first follow-up survey (T1) by asking participants if they had been arrested. Incident arrest was defined as arrest at follow-up, while subjects with lifetime arrest at baseline were excluded from the analyses.

### Baseline dimensional symptom scales

Symptoms of all baseline mental disorders were assessed at baseline using the respective CIDI sections. For each mental disorder, including substance use disorders (32 items), bipolar disorder (17 items), major depression/dysthymia (28 items), anxiety disorders (43 items), attention deficit hyperactivity disorder (9 items), oppositional defiant disorder (11 items) and conduct disorder (11 items), dimensional symptom scales were constructed by summing the participant’s binary scores of the individual items of the respective CIDI sections. In addition, a dimensional scale was constructed for antisocial personality by summing the individual items regarding antisocial personality using questions from the International Personality Disorder Examination (7 items). Thus, eight continuous symptom scales were generated.

### Childhood trauma

Childhood trauma was assessed at baseline by asking participants about physical, psychological or sexual abuse before the age of 16 years. Conform previous analyses [20, 44], childhood trauma was defined present if a participant had experienced psychological abuse on two or more occasions, or physical abuse / sexual abuse on one or more occasion.

### Social support

Social support from three resources (partner, family or friends, neighbours) was assessed at T1 with two questions on instrumental and emotional support from each of these resources in the direct network. These referred to the extent participants could rely on them for help if they had a problem and could open up to them if they needed to talk about worries. The four response categories ranged from ‘not at all’ to ‘a lot’. The mean score on both questions was used to generate individual scores of perceived social support score for each individual resource, taking the participant’s evaluation of it into account. A social support variable was then calculated as the mean of these three scores (or two if a participant did not have a partner at the time of the interview).

### Negative life events

The presence of negative life events in the previous 12 months was measured at baseline. Negative life events were based on the Brugha life events categories [45] and included 10 different events, for example death of a relative, divorce and major financial difficulties. The numbers of events were categorized as 0, 1 and more than 1, in order to construct three approximately equal groups.

### Statistical analyses

Statistical analyses were performed using Stata version 13 [46]. Only participants with complete data were analysed. First, baseline characteristics of all dependent and independent variables were assessed for the complete sample. Moreover, subjects with clinically validated PE at baseline were compared with subjects without clinically validated PE using chi-square tests and independent sample t-tests.

Second, logistic regression analyses were performed in the complete sample using self-reported or clinically validated PE as the independent variable and the different outcomes (i.e. physical violence, psychological violence or arrest) as the dependent variables. The independent variables were divided into hallucinations and delusions in separate analyses. Logistic regression analyses were performed in four different models with varying levels of adjustment. In the first model, the unadjusted associations between the various exposures and outcome measures were examined. The second model added sociodemographic characteristics as independent variables. In the third model, associations were adjusted for sociodemographic characteristics and dimensional symptom scales for substance abuse, bipolar disorder, major depression/dysthymia, anxiety disorders, ADHD, ODD, conduct disorder, antisocial personality. Before adding the symptom scores, linearity of the associations was assessed by adding both the linear and the quadratic term of the symptoms scale in separate logistic regression analyses. If the quadratic term reached statistical significance, both the quadratic and the linear term were used; if the quadratic term did not reach significance, only the linear terms were used. In the fourth and final model, contextual factors including childhood trauma, negative life events and social support were added to the regression models.

Third, dose-response associations between PE and various outcomes were examined by repeating analyses using a continuous scale of PE as the independent variable. Finally, confounding by presence of categorical mental disorder was examined by repeating all analyses stratified by presence or absence of any baseline mental disorder.

## Results

### Baseline characteristics

Of the included participants, 10.4% (N = 630) had self-reported PE and 6.2% (N = 362) had clinically validated PE, after exclusion of individuals with psychotic disorder (n = 43). Compared with the group without clinically validated PE, the group with clinically validated PE was significantly younger, had a smaller proportion of males and higher scores on all symptom scales (Table 1). Moreover, at baseline, lifetime prevalence of both physical and psychological violence perpetration towards intimate partners was higher among individuals with PE; physical violence perpetration towards children was more prevalent as well. In addition, individuals with PE had significantly more negative life events, including childhood trauma, and experienced less social support.

**Table 1:** Baseline characteristics.

	Complete sample	Subjects with PE*	Subjects without PE*	t	χ^2^	df	p **
*Demographics*:							
N	6088	362	5460	-	-	-	-
Number of males (%)	2755 (45.3)	127 (35.1)	2511 (46.0)	-	16.30	1	<0.001
Age (SD)	44.3 (12.6)	43.1 (13.1)	44.5 (12.5)	2.00	-	5820	0.046
*Baseline violence/arrests*:							
Physical violence towards intimate partner, N (%)	324 (5.7)	43 (12.8)	250 (4.9)	-	38.82	1	<0.001
Psychological violence towards intimate partner, N (%)	1353 (25.2)	132 (41.5)	1135 (23.5)	-	52.02	1	<0.001
Physical violence towards children, N (%)	113 (2.7)	12 (5.1)	90 (2.4)	-	6.83	1	0.009
Psychological violence towards children, N (%)	616 (15.3)	39 (18.0)	544 (14.8)	-	1.59	1	0.208
Ever arrested, N (%)	1301 (21.4)	90 (24.9)	1138 (20.9)	-	3.39	1	0.066
*Baseline symptom dimensions*:							
Mean number of depressive symptoms, N (SD)	3.8 (7.2)	7.8 (9.4)	3.4 (6.8)	-11.67	-	5820	<0.001
Mean number of anxiety symptoms, N (SD)	4.7 (6.5)	9.2 (8.5)	4.2 (6.1)	-14.70	-	5820	<0.001
Mean number of mania symptoms, N (SD)	0.6 (1.7)	1.6 (1.6)	0.5 (1.4)	-13.09	-	5819	<0.001
Mean number of substance use disorder symptoms, N (SD)	1.6 (2.1)	2.5 (2.9)	1.5 (1.8)	-9.99	-	5820	<0.001
Mean number of ADHD symptoms, N (SD)	0.6 (2.8)	1.5 (4.6)	0.5 (2.6)	-6.15	-	5506	<0.001
Mean number of ODD symptoms, N (SD)	0.1 (0.7)	0.3 (1.0)	0.1 (0.6)	-4.63	-	5690	<0.001
Mean number of conduct disorder symptoms, N (SD)	0.3 (0.9)	0.5 (1.2)	0.2 (0.8)	-5.64	-	5820	<0.001
Mean number of antisocial personality symptoms, N (SD)	2.1 (0.9)	2.4 (1.0)	2.1 (0.9)	-6.17	-	5820	<0.001
*Baseline other confounders*:							
Childhood trauma, N (%)	1601 (26.9)	188 (52.5)	1290 (24.2)	-	140.43	1	<0.001
Mean number of negative life-even past 12 months, N (%)	0.7 (0.8)	1.0 (0.8)	0.7 (0.8)	-8.65	-	5696	<0.001
Social support score, N (SD)	3.4 (0.6)	3.2 (0.7)	3.4 (0.6)	5.08	-	4615	<0.001

*Clinically validated PE

**p-value resulting from t-test or chi-square test for difference between participants with vs. without PE

### Physical violence

Incidence of physical violence was 1.3% (N = 36). Individuals with any self-reported or clinically validated PE were at increased risk of physical violence with odds ratios (OR) of 2.63 (95% CI 1.15–6.03; self-reported PE) and 2.80 (95% CI 1.08–7.30; clinically validated PE), respectively (Table 2). Dividing PE into hallucinations and delusions revealed that delusions were not associated with an increased risk of violence. On the other hand, both self-reported and clinically validated hallucinations at baseline were associated with physical violence at follow-up. Adjusting the analyses for demographic characteristics (model 2) increased the ORs. Further adjustment for dimensional symptom scales (model 3) slightly decreased the associations, but hallucinations remained associated with an increased risk of physical violence. In the final model, associations further decreased. However, self-reported (OR = 2.83, 95% CI 1.05–7.65) and clinically validated hallucinations (OR = 3.59, 95% CI 1.09–11.81) remained significantly associated with physical violence perpetration.

**Table 2:** Results (odds ratios and 95% CI) from logistic regression analyses on the association between self-reported (SR) and clinically validated (CV) psychotic experiences and six-year incidence of physical violence perpetration in a general population sample and two subsamples.

	Incident physical violence			
	Model 1^a^	Model 2^b^	Model 3^c^	Model 4^d^
	**Complete sample (excluding baseline psychotic disorder; N = 3283)**			
*SR*				
Any PE	2.63 (1.15–6.03)*	2.27 (0.91–5.65)	1.83 (0.67–5.04)	1.54 (0.55–4.37)
Any delusion	2.34 (0.82–6.70)	1.64 (0.48–5.55)	1.06 (0.27–4.23)	0.93–0.23–3.74)
Any hallucination	3.50 (1.44–8.50)**	3.75 (1.49–9.47)**	3.37 (1.28–8.90)*	2.83 (1.05–7.65)*
*CV*				
Any PE	2.80 (1.08–7.30)*	2.96 (1.11–7.91)*	2.44 (0.83–7.19)	2.13 (0.71–6.41)
Any delusion	1.51 (0.36–6.37)	1.59 (0.37–6.87)	1.14 (0.23–5.59)	1.04 (0.21–5.27)
Any hallucination	3.76 (1.30–10.86)*	4.42 (1.48–13.20)**	4.34 (1.36–13.86)*	3.59 (1.09–11.81)*
	**Subsample of individuals without any baseline mental disorder (N = 2014)**			
*SR*				
Any PE	1.11 (0.15–8.46)	1.61 (0.20–12.81)	1.25 (0.13–11.64)	1.00 (0.10–10.15)
Any delusion	N.A.	N.A.	N.A.	N.A.
Any hallucination	1.73 (0.23–13.29)	2.27 (0.28–18.48)	1.72 (0.17–17.30)	1.34 (0.12–14.98)
*CV*				
Any PE	1.76 (0.23–13.49)	2.96 (0.36–23.99)	1.88 (0.19–19.07)	1.55 (0.14–17.58)
Any delusion	N.A.	N.A.	N.A.	N.A.
Any hallucination	2.83 (0.37–21.89)	4.90 (0.59–40.97)	3.08 (0.26–36.80)	2.37 (0.18–32.11)
	**Subsample of individuals with any baseline mental disorder (excluding baseline psychotic disorder, N = 1269)**			
*SR*				
Any PE	2.78 (1.07–7.25)*	2.57 (0.88–7.48)	2.84 (0.89–9.00)	2.36 (0.72–7.75)
Any delusion	2.57 (0.85–7.77)	2.00 (0.56–7.22)	2.06 (0.52–8.27)	1.75 (0.43–7.12)
Any hallucination	3.60 (1.29–10.02)*	4.48 (1.19–13.48)**	5.30 (1.67–16.84)**	4.49 (1.37–17.73)*
*CV*				
Any PE	2.64 (0.86–8.07)	2.99 (0.94–9.54)	3.48 (0.99–12.29)	3.03 (0.84–11.00)
Any delusion	1.63 (0.37–7.20)	1.84 (0.41–8.38)	1.82 (0.35–9.39)	1.58 (0.29–8.42)
Any hallucination	3.44 (0.97–12.16)	4.61 (1.22–17.38)*	6.15 (1.48–25.60)*	5.22 (1.22–22.37)*

* p<0.05,

** p<0.01

^a^Model 1: Unadjusted

^b^Model 2: Adjusted for demographics (gender, age, ethnicity, education, employment, socioeconomic status)

^c^Model 3: Adjusted for demographics and dimensional psychopathology (substance abuse, bipolar disorder, major depression, dysthymia, anxiety disorders, ADHD, ODD, conduct disorder, antisocial personality)

^d^Model 4: Adjusted for demographics, dimensional psychopathology, contextual factors (childhood trauma, social support, negative life events)

Continuously defined PE were similarly associated with physical violence perpetration as dichotomously defined PE. In the fully adjusted model, the risk of physical violence perpetration increased in a dose-response fashion as the number of clinically validated hallucinations increased (OR = 2.71, 95% CI 1.39–5.28), but there was no association between number of delusions and physical violence perpetration.

In the subsample without baseline mental disorder, there was no association between PE and physical violence perpetration (Table 2). However, in the subgroup of individuals with any baseline mental disorder, individuals with PE were at increased risk of physical violence perpetration. Analysing hallucinations and delusions separately revealed that both self-reported hallucinations (OR = 4.49. 95% CI 1.37–17.73) and clinically validated hallucinations (OR = 5.22, 95% CI 1.22–22.37) were associated with physical violence perpetration, even after adjustment for demographics, dimensional psychopathology and contextual confounders (Table 2). Similar to the results in the complete sample, there was no association between delusions and physical violence perpetration in the subgroup of individuals with any baseline mental disorder.

### Psychological violence

Incidence of psychological violence was 20.1% (N = 265). In the complete sample, both delusions and hallucinations at baseline were associated with an increased risk of psychological violence perpetration at follow-up (Table 3). Adjusting the analyses for demographical characteristics did not change the results (model 2). However, correcting for dimensional psychopathology (model 3) decreased the associations and only clinically validated hallucinations remained significantly associated with psychological violence perpetration. However, inclusion of contextual confounders in the final model further decreased the ORs and no association remained. Results of the analyses using a continuous scale of PE were similar to the results using dichotomously defined PE as the independent variable.

**Table 3:** Results (odds ratios and 95% CI) from logistic regression analyses on the association between self-reported (SR) and clinically validated (CV) psychotic experiences and six-year incidence of psychological violence in a general population sample and two subsamples.

	Incident psychological violence			
	Model 1^a^	Model 2^b^	Model 3^c^	Model 4^d^
	**Complete sample (excluding baseline psychotic disorder; N = 3283)**			
*SR*				
Any PE	1.91 (1.33–2.73)**	2.18 (1.47–3.23)**	1.51 (0.94–2.41)	1.21 (0.74–1.97)
Any delusion	2.13 (1.38–3.29)**	2.35 (1.47–3.74)**	1.61 (0.92–2.81)	1.34 (0.76–2.38)
Any hallucination	1.97 (1.29–2.99)**	2.13 (1.34–3.39)**	1.52 (0.88–2.60)	1.17 (0.67–2.06)
*CV*				
Any PE	1.91 (1.25–2.91)**	2.37 (1.50–3.76)**	1.74 (1.01–3.00)*	1.40 (0.79–2.48)
Any delusion	1.63 (0.96–2.77)	2.00 (1.13–3.55)*	1.39 (0.69–2.80)	1.20 (0.59–2.47)
Any hallucination	2.32 (1.40–3.84)**	2.70 (1.55–4.69)**	2.02 (1.06–3.85)*	1.56 (0.79–3.06)
	**Subsample of individuals without any baseline mental disorder (N = 2014)**			
*SR*				
Any PE	1.49 (0.72–3.12)	1.84 (0.83–4.09)	1.96 (0.79–4.84)	1.54 (0.59–4.01)
Any delusion	2.56 (1.01–6.49)*	3.20 (1.18–8.65)*	3.50 (1.09–11.28)*	3.18 (0.96–10.60)
Any hallucination	1.03 (0.38–2.75)	1.17 (0.41–3.33)	1.21 (0.38–3.89)	0.79 (0.22–2.92)
*CV*				
Any PE	1.10 (0.41–2.97)	1.54 (0.53–4.48)	2.06 (0.65–6.54)	1.56 (0.44–5.58)
Any delusion	0.85 (0.19–3.85)	1.17 (0.24–5.75)	1.71 (0.32–9.14)	1.76 (0.31–9.85)
Any hallucination	1.14 (0.32–4.06)	1.65 (0.42–6.47)	2.04 (0.47–8.89)	1.13 (0.19–6.63)
	**Subsample of individuals with any baseline mental disorder (excluding baseline psychotic disorder, N = 1269)**			
*SR*				
Any PE	1.76 (1.15–2.69)**	1.98 (1.23–3.18)**	1.48 (0.84–2.61)	1.23 (0.68–2.21)
Any delusion	1.68 (1.02–2.76)*	1.78 (1.04–3.05)*	1.47 (0.78–2.79)	1.21 (0.62–2.33)
Any hallucination	2.01 (1.24–3.27)**	2.14 (1.25–3.68)**	1.53 (0.81–2.91)	1.31 (0.68–2.52)
*CV*				
Any PE	1.86 (1.14–3.02)*	2.26 (1.32–3.85)**	1.77 (0.92–3.40)	1.55 (0.80–2.98)
Any delusion	1.50 (0.84–2.68)	1.83 (0.97–3.45)	1.57 (0.72–3.44)	1.28 (0.58–2.83)
Any hallucination	2.32 (1.30–4.11)**	2.50 (1.34–4.68)**	1.87 (0.88–3.99)	1.74 (0.81–3.71)

* p<0.05,

** p<0.01

^a^Model 1: Unadjusted

^b^Model 2: Adjusted for demographics (gender, age, ethnicity, education, employment, socioeconomic status)

^c^Model 3: Adjusted for demographics and dimensional psychopathology (substance abuse, bipolar disorder, major depression, dysthymia, anxiety disorders, ADHD, ODD, conduct disorder, antisocial personality)

^d^Model 4: Adjusted for demographics, dimensional psychopathology, contextual factors (childhood trauma, social support, negative life events)

In the subsample of individuals without any mental disorder at baseline, self-reported delusions were associated with an increased risk of psychological violence perpetration (Table 3). However, in the fully adjusted model, self-reported delusions were no longer associated with an increased risk of psychological violence perpetration, neither was there an association between clinically validated delusions and psychological violence perpetration. Results of the analyses in the subsample of individuals with any baseline mental disorder were similar to the results in the complete sample.

### Arrest

Incidence of arrest was 2.6% (N = 99). Individuals with self-reported or clinically validated PE at baseline were at increased risk of incident arrest (Table 4). Further assessment of this association showed that delusions were associated with an increased risk of arrest at follow-up, while hallucinations were not. Adjustment for demographics, dimensional psychopathology and contextual confounders slightly decreased the effect sizes, but both self-reported delusions (OR = 3.06 95% CI 1.55–6.03) and clinically validated delusions (OR = 3.24 95% CI 1.47–7.13) remained significantly associated with incident arrest.

**Table 4:** Results (odds ratios and 95% CI) from logistic regression analyses on the association between self-reported (SR) and clinically validated (CV) psychotic experiences and three-year incidence of arrest in a general population sample and two subsamples.

	Incident arrest			
	Model 1^a^	Model 2^b^	Model 3^c^	Model 4^d^
	**Complete sample (excluding baseline psychotic disorder; N = 4218)**			
*SR*				
Any PE	3.16 (1.96–5.11**	3.27 (1.94–5.51)**	2.40 (1.28–4.49)**	2.12 (1.12–4.01)*
Any delusion	4.50 (2.70–7.49)**	4.49 (2.57–7.85)**	3.52 (1.79–6.90)**	3.06 (1.55–6.03)**
Any hallucination	1.92 (0.98–3.74)	2.01 (1.00–4.07)	1.44 (0.63–3.29)	1.22 (0.52–2.84)
*CV*				
Any PE	3.07 (1.74–5.44)**	3.65 (1.98–6.71)**	2.47 (1.17–5.20)*	2.18 (1.03–4.64)*
Any delusion	4.04 (2.20–7.44)**	4.84 (2.51–9.33)**	3.71 (1.70–8.14)**	3.24 (1.47–7.13)**
Any hallucination	1.75 (0.75–4.07)	2.06 (0.85–4.99)	1.03 (0.33–3.21)	0.86 (0.27–2.73)
	**Subsample of individuals without any baseline mental disorder (N = 2536)**			
*SR*				
Any PE	3.47 (1.51–7.98)**	3.62 (1.37–9.54)**	3.43 (1.18–9.98)*	2.76 (0.93–8.23)
Any delusion	6.59 (2.82–15.38)**	8.06 (2.96–21.99)**	7.48 (2.46–22.78)**	6.25 (2.01–19.44)**
Any hallucination	0.68 (0.09–4.98)	0.69 (0.09–5.44)	0.65 (0.07–5.76)	0.43 (0.04–4.20)
*CV*				
Any PE	3.23 (1.12–9.31)*	4.31 (1.35–13.76)*	4.28 (1.25–14.67)*	3.30 (0.92–11.81)
Any delusion	5.62 (1.91–16.48)**	8.23 (2.48–27.34)**	6.96 (1.98–24.54)**	5.37 (1.45–19.79)*
Any hallucination	1.10 (0.15–8.15)	1.28 (0.15–10.62)	1.15 (0.11–11.87)	0.79 (0.07–9.15)
	**Subsample of individuals with any baseline mental disorder (excluding baseline psychotic disorder, N = 1682)**			
*SR*				
Any PE	2.48 (1.36–4.49)**	2.37 (1.25–4.49)**	1.98 (0.89–4.38)	1.81 (0.80–4.08)
Any delusion	3.04 (1.60–5.78)**	2.70 (1.36–5.35)**	2.50 (1.07–5.87)*	2.25 (0.94–5.36)
Any hallucination	2.00 (0.96–4.18)	1.97 (0.90–4.28)	1.72 (0.68–4.37)	1.55 (0.60–3.99)
*CV*				
Any PE	2.42 (1.22–4.81)*	2.59 (1.25–5.37)*	1.95 (0.77–4.96)	1.86 (0.72–4.77)
Any delusion	2.80 (1.33–5.91)**	3.09 (1.39–6.83)**	2.88 (1.06–7.80)*	2.72 (1.00–7.44)
Any hallucination	1.63 (0.63–4.20)	1.73 (0.64–4.67)	0.92 (0.24–3.54)	0.84 (0.21–3.28)

* p<0.05,

** p<0.01

^a^Model 1: Unadjusted

^b^Model 2: Adjusted for demographics (gender, age, ethnicity, education, employment, socioeconomic status)

^c^Model 3: Adjusted for demographics and dimensional psychopathology (substance abuse, bipolar disorder, major depression, dysthymia, anxiety disorders, ADHD, ODD, conduct disorder, antisocial personality)

^d^Model 4: Adjusted for demographics, dimensional psychopathology, contextual factors (childhood trauma, social support, negative life events)

Associations between continuously defined PE and arrest were similar to the associations between dichotomously defined PE and arrest. In the fully adjusted model, the risk of arrest increased in a dose-response fashion as the number of clinically validated delusions increased (OR = 1.57, 95% CI 1.00–2.47). There was no association between the number of hallucinations and risk of arrest.

Analyses in the subsample of individuals without any baseline mental disorder showed that both self-reported delusions (OR = 6.25, 95% CI 2.01–19.44) and clinically validated delusions (OR = 5.37, 95% CI 1.45–19.79) were strongly associated with incident arrest in the fully adjusted model (Table 4). Analyses in the sample with any baseline mental disorder showed similar results, although results were no longer statistically significant in the final model (Table 4).

## Discussion

### Overview of results

The present study is the first to investigate the longitudinal association between both self-reported and clinically validated PE on the one hand, and incident physical violence, psychological violence and arrest on the other, in a prospective, general population cohort. Participants with hallucinations at baseline were at increased risk of perpetrating physical violence over the follow-up period of six years, even after adjustment for demographics, dimensional psychopathology and contextual confounders. Delusions were not associated with an increased risk of physical violence in the complete sample. On the other hand, both delusions and hallucinations were associated with an increased risk of psychological violence in the unadjusted analyses. However, adjustment for dimensional psychopathology decreased all effect sizes and rendered them statistically non-significant, indicating that the association between PE and psychological violence can be explained by the presence of non-psychotic psychopathology. The finding that PE in the subsample without any mental disorder at baseline did not predict psychological violence, even in the unadjusted analyses, supports this explanation. Finally, the presence of delusions at baseline was associated with an increased risk of reporting arrest, after controlling for demographics, dimensional psychopathology and contextual risk factors. Hallucinations were not associated with an increased risk of arrest. Analyses using continuous scales of PE showed similar associations as the analyses using dichotomously defined PE. The risk of physical violence perpetration increased as the number of hallucinations increased; the risk of arrest increased as the number of delusions increased.

### Psychotic experiences and physical violence perpetration

Previous research suggested that a specific subset of delusional symptoms called ‘threat/control-override’ (TCO) symptoms is associated with violence perpetration, because these symptoms cause the person to believe that people are out to harm them or that outside forces are controlling their minds [6, 47, 48]. The present study cannot be considered as a replication, as the results showed that hallucinations were associated with an increased risk of physical violence perpetration, while delusions were not associated with violence perpetration. This finding is consistent with previous studies in the general population that also reported associations between hallucinations and physical violence perpetration [3–5]. However, a recent meta-analysis of seven cross-sectional population surveys found no association between hallucinations and violence; only paranoid ideation was associated with an increased risk of violence perpetration [7]. In the present study, delusions predicted violence perpetration only in the subsample with any baseline mental disorder, although results were statistically non-significant. Thus, PE in the context of a mental disorder may be differently associated with physical violence than PE in absence of a mental disorder. However, more research is required to replicate this finding. Moreover, a previous prospective study showed that delusions among psychiatric inpatients after discharge did not predict future violence, unless there was a close temporal relationship between delusional ideation and violence [49]. In the present analyses, this temporal proximity between PE and violence could not be taken into account since at baseline only lifetime PE were assessed and at follow-up only violence in the six years before the interview was assessed. This may explain why delusions were not associated with future physical violence in the analyses in the complete sample.

Although the association between PE and physical violence perpetration has been confirmed in various cross-sectional studies, the mechanism behind this association remains unclear. The association between PE and violence may be the result of confounding by other variables. Previous research showed that the association between PE and auto-aggressive behaviour (i.e. suicidal behaviour and self-injury) is confounded by co-occurring non-psychotic psychopathology [50], thus making it likely that this also applies to hetero-aggression (i.e. interpersonal violence), as confirmed in the study by Coid and colleagues [7]. The present analyses were adjusted for demographics, dimensional non-psychotic psychopathology and several contextual confounders identified in previous studies [20] which included childhood trauma, negative life events and social support. Consistent with previous research [20], adjustment for these variables decreased effect sizes. This indicates that the associations are at least in part confounded. Inclusion of other variables that are associated with both PE and violence perpetration in the regression models, for example stress reactivity [51], anger [3] or impulsivity [52] might have further decreased effect sizes.

Moreover, the present analyses were only adjusted for baseline non-psychotic psychopathology. However, individuals with PE are at increased risk to develop a mental disorder [1, 27, 53] and, thus, the reported association between hallucinations and violence perpetration can be explained by non-psychotic psychopathology after baseline. In other words, PE and non-psychotic psychopathology may lie on the causal pathway towards the outcome of violence or arrest. To examine this, sensitivity analyses were conducted, which showed that adjustment for incident mental disorders did not decrease ORs. On the other hand, ORs increased when a sensitivity analysis was conducted in the subsample of individuals with incident mental disorders. This finding is consistent with the main results in Table 2 that showed that hallucinations were particularly predictive of violence perpetration in the context of a baseline mental disorder. Thus, the association between hallucinations and violence perpetration possibly reflects the increased risk of violence perpetration in individuals in distress in the context of a mental disorder. This is in agreement with previous studies that demonstrated that PE serve as an indicator of illness severity and poor outcome in persons with non-psychotic psychopathology [27, 38, 54–56].

In addition, it is possible that the association between hallucinations and violence perpetration reflects the association between one specific PE, for example auditory hallucinations, and violence perpetration, since previous, cross-sectional work found an association between command hallucinations and violence perpetration [57]. More longitudinal research with larger sample sizes is required to investigate this question, since statistical power in the current dataset is too low to assess associations between specific PE and violence perpetration.

### Psychotic experiences and psychological violence

The present study provides the first evidence that PE in the general population are prospectively associated with an increased risk of psychological violence perpetration. However, after adjustment for dimensional psychopathology, no association remained, indicating that the association between PE and psychological violence is non-specific and reflects the increased risk of psychological violence in individuals with non-psychotic psychopathology [20] that is prevalent among individuals with PE [27–29].

### Psychotic experiences and arrest

Incident arrest was more frequent in individuals experiencing delusions than in individuals without delusions. This result was found in both the complete sample and the subsample without baseline mental disorders, even in the most adjusted models. Contrary to the association found between hallucinations and physical violence perpetration, there was no association between hallucinations and arrest. As far as we are aware, only one previous study assessed the association between PE and arrest and showed that PE were associated with an increased risk of arrest for both aggravated assault and other illegal behaviours [4]. On the other hand, previous studies showed that the prevalence of psychotic disorder is much higher among jail detainees than among general population samples in the same age group [58], indicating that there is an association between full blown psychosis and arrest. Similarly, Vreugdenhil and colleagues showed that 34% of a sample of incarcerated boys reported any psychotic symptom [24]. Our results indicate that low grade PE, as part of the extended psychosis phenotype [28], are also associated with an increased risk of arrest. The finding that delusions predict arrest is consistent with previous work that demonstrated that paranoid ideation is associated with violent incidents involving the police [7].

Sensitivity analyses were conducted to examine the mediating effect of incident mental disorders on the association between delusions and arrest. Results showed that inclusion of incident mental disorders in the regression models increased ORs. Moreover, ORs increased further when sensitivity analyses were conducted in the subsample without incident mental disorders. This result is consistent with the main results in Table 4 that showed that delusions were particularly predictive of arrest in the absence of a mental disorder. It is unclear why hallucinations are particularly predictive of violence perpetration in the context of a mental disorder, while delusions are particularly predictive of arrest in the absence of a mental disorder. Moreover, it remains unclear why physical violence perpetration is predicted by hallucinations, while arrest is best predicted by delusions. A possible explanation is that the outcome of arrest does not only include arrests for physical violence but also for other behaviours or criminal offenses that may be associated with delusions instead of hallucinations—for example social conflicts in the context of disordered behaviour arising from paranoid or grandiose delusions. More longitudinal research is needed to replicate the results of the present study and examine underlying mechanisms.

### Self-reported versus clinically validated psychotic experiences

The results of this study show that both self-reported and clinically validated PE are predictive of incident physical violence, psychological violence and arrest. In general, ORs for the associations between clinically validated PE and the various outcomes were slightly larger than effect sizes for self-reported PE. This finding is consistent with previous work that showed that clinically validated PE were stronger associated with various mental health outcomes than self-reported PE [41].

### Strengths and limitations

Strength of the present study is the prospective, longitudinal study design that enables the assessment of the temporal sequence of the association between PE and violence, while previous studies were cross-sectional. Another strength is the use of both self-reported and clinically validated PE. Previous studies on the association between PE and violence only included self-reported PE as exposure. A final strength is the use of dimensional symptom scales to adjust the regression models, rather than using a narrow, dichotomous definition of mental disorder that leaves room for residual confounding.

The results of the present study should be interpreted in the light of some methodological limitations. First, the outcome of physical violence was rare. Even though the study included 6646 individuals at baseline, statistical power was low, as shown by the wide 95% confidence intervals. For that reason, it was impossible to assess the association between individual PE items and the various outcomes. Second, the questions used to assess physical violence and psychological violence at follow-up were not identical to the questions used at baseline, since at baseline only violence towards an intimate partner or children was assessed. To overcome this limitation, individuals who never had a partner or children at baseline were excluded from the analyses for the violence outcomes. However, violence perpetration against anyone other than a partner or child could still be missed at baseline. Fortunately, the assessment of baseline physical violence could be improved by integrating an additional question from the screening section of the CIDI. Moreover, research shows that violence perpetration often targets intimate partners [59]. It is therefore unlikely that the discrepancy between the baseline and follow-up assessments of violence perpetration influences the results to a great extent. Third, not every dimensional psychopathology scale could be generated for each participant, since impulse-control disorders were only assessed in subjects aged 18–44 years and other mental disorders only if a participant answered positively to a key question for the respective disorder in the screening section. This may have resulted in an overestimation of reported associations. Finally, follow-up data was missing for a proportion of the cohort (31%). Attrition at follow-up was not associated with baseline mental health status [60], PE or any violence perpetration. However, baseline arrest was weakly associated with an increased risk of participant attrition (OR = 1.23, 95% CI 1.09–1.40). Since individuals with baseline arrest were also at increased risk for violence perpetration, this may have resulted in more conservative rather than spurious associations.

### Future research

The present study provides fresh evidence that both self-reported and clinically validated PE in general population individuals are associated with an increased risk of future violence perpetration and arrest. However, more longitudinal research with larger samples sizes is required to confirm these findings. The use of larger samples offers the opportunity to assess the association between individual PE and violence and arrest and thus to examine whether there is any association between the aforementioned TCO symptoms and perpetration of violence in the general population. The results showed that self-reported PE can be used as a valid measure to study these associations. Moreover, future research should take the temporal proximity of exposures and outcomes into account in order to assess whether this changes the associations between PE and physical violence, psychological violence and arrest as reported in this study.
